# Linking cortical visual processing to viewing behavior using fMRI

**DOI:** 10.3389/fnsys.2013.00109

**Published:** 2013-12-18

**Authors:** Jan Bernard C. Marsman, Remco Renken, Koen V. Haak, Frans W. Cornelissen

**Affiliations:** ^1^NeuroImaging Center, University Medical Center GroningenGroningen, Netherlands; ^2^Laboratory for Experimental Ophthalmology, University Medical Center GroningenGroningen, Netherlands; ^3^Radboud University Nijmegen, Donders Institute for Brain, Cognition and BehaviourNijmegen, Netherlands

**Keywords:** eye movements, fMRI, fixation-based event related fMRI, natural viewing behavior, dorsal stream, ventral stream, independent component analysis, scene perception

## Abstract

One characteristic of natural visual behavior in humans is the frequent shifting of eye position. It has been argued that the characteristics of these eye movements can be used to distinguish between distinct modes of visual processing (Unema et al., 2005). These viewing modes would be distinguishable on the basis of the eye-movement parameters fixation duration and saccade amplitude and have been hypothesized to reflect the differential involvement of dorsal and ventral systems in saccade planning and information processing. According to this hypothesis, on the one hand, while in a “pre-attentive” or ambient mode, primarily scanning eye movements are made; in this mode fixation are relatively brief and saccades tends to be relatively large. On the other hand, in “attentive” focal mode, fixations last longer and saccades are relatively small, and result in viewing behavior which could be described as detailed inspection. Thus far, no neuroscientific basis exists to support the idea that such distinct viewing modes are indeed linked to processing in distinct cortical regions. Here, we used fixation-based event-related (FIBER) fMRI in combination with independent component analysis (ICA) to investigate the neural correlates of these viewing modes. While we find robust eye-movement-related activations, our results do not support the theory that the above mentioned viewing modes modulate dorsal and ventral processing. Instead, further analyses revealed that eye-movement characteristics such as saccade amplitude and fixation duration did differentially modulate activity in three clusters in early, ventromedial and ventrolateral visual cortex. In summary, we conclude that evaluating viewing behavior is crucial for unraveling cortical processing in natural vision.

## Introduction

In daily life, we make numerous eye movements. This natural viewing behavior of human observers has been characterized and studied extensively. One of the first and most famous studies is by Alfred Yarbus, who showed that human eye movement behavior depends upon task context and stimulus content (Yarbus, 1967). Since, numerous studies have confirmed this aspect of human viewing behavior (e.g., Rothkopf et al., 2007).

Unema et al. (2005) reported another aspect of human viewing behavior. Following the presentation of a novel scene, observers initially scan the scene by quickly making a series of relatively large saccadic eye movements. Each of these large-amplitude saccades is followed by a relatively brief fixation, enabling the observer to cover a large image region in the first few seconds of a presentation. Over time, the average duration of the fixations increases, while at the same time the average saccadic amplitude decreases. Such longer fixations in combination with small-amplitude saccadic eye movements allow for a more detailed inspection of scene elements (Antes, 1974; Unema et al., 2005; Over et al., 2007; Pannasch et al., 2008).

This behavior has been interpreted to imply that people build-up some sort of spatial map by quickly visiting key elements in the scene for further analysis at a later stage. This hypothesis is in line with findings of studies on scene perception (Fize et al., 2000; Rensink, 2004). In only a few milliseconds, the gist of a scene can be extracted in order to determine salient objects, which are then quickly scanned during an initial series of brief fixations. Over time, fixation duration increases to allow for more detailed inspection of specific elements in the scene.

Many lines of evidence suggest two separate information streams project from V1 into other brain regions (Ingle et al., 1967; Milner and Goodale, 1993, 2008; Velichkovsky, 2002). One stream—referred to as the ventral or “what” stream—projects toward temporal areas of the brain and is involved in object analysis (Milner and Goodale, 2008). The second stream—referred as the dorsal or “where” stream—projects to parietal areas and deals with spatial vision. Based on the previously described eye-movement findings, it has been suggested that natural viewing behavior can be categorized into two distinct types of viewing behavior that are associated with processing in the dorsal and the ventral pathways (Velichkovsky, 2002; Unema et al., 2005). Preattentive scanning behavior, evident from large saccades combined with short fixations, would reflect dorsal pathway processing. In contrast, attentive inspection behavior, evident from small saccades combined with long duration fixations, would reflect ventral pathway processing. Whether and how these two different types of viewing behavior indeed imply the involvement of these distinct neural systems is—at present—not known.

Here, we use combined eyetracking and fMRI to investigate the neural correlates of the different types of viewing behavior. Specifically, we test the hypothesis that short fixations coupled with large saccadic amplitudes—which would be related to the build-up of a spatial map—reflect dorsal stream processing. In contrast, longer fixations coupled with small saccades would show more activity in regions along the ventral visual stream.

## Methods

### Subjects

Sixteen healthy right-handed subjects (three of whom were female) were scanned in a Philips 3 Tesla Intera MRI scanner (Philips, Best, The Netherlands). All subjects maintained normal healthy vision. All subjects gave informed consent and ethical approval was provided by the local medical ethical committee.

### Stimuli

**Figure 1:**
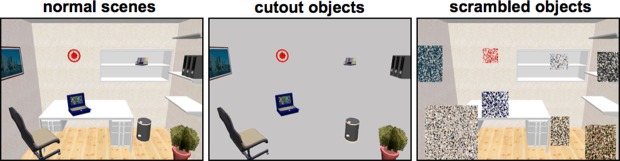
**Example stimuli used in the experiment**. Stimuli from the study by Unema et al. (2005) were used and adapted into two additional variants (cut-out objects and scrambled objects).

Stimuli were taken from the original eye tracking study by Unema et al. (2005) and consisted of 12 computer generated indoor scenes, each containing eight household objects (hereafter referred to as “normal scenes”). Furthermore, we created two additional sets by manipulating the original images (Figure 1): one set in which the background was removed, so that only the objects are visible on a solid grey background (“cutout objects”). In the other set the objects were scrambled, leaving the scene’s background intact (“scrambled objects”). This scrambling was performed by rasterizing a square patch the size of each object in patches of 5 × 5 pixels, and shuffling these patches across the raster. Images were 800 × 600 pixels and were displayed on a translucent display positioned at the head-end of the fMRI scanner using a video projector (Barco, Kortrijk, Belgium) with a resolution of 1024 × 768 pixels. Participants viewed the screen via a mirror. The distance from the eyes to the screen was 75 cm, and the width and height of the translucent display was 44 and 34 cm, respectively. This subtends a visual angle of 32 × 25.5° for the entire screen. The stimuli were not presented in full-screen, due to known eye-tracking difficulties in the upper and lower corners of the screen: The corneal reflection would fall behind the lower eyelid when subjects would be looking entirely upwards. Moreover, when looking entirely downward, subjects tended to close their eyes more, which also resulted in loss of eye tracking. Therefore, the visual angle of the stimuli subtended 25 × 20°. Each stimulus was shown for 10 s.

### Experimental paradigm

Participants were instructed to perform normal viewing behavior during the experiment. After each set of four stimuli a fixation cross was shown for 10 s. Four functional runs and one anatomical scan was recorded (see the “functional imaging” section). During the first and third run the stimuli from “normal scenes” and “cutout objects” were displayed. During the other two runs, subjects were presented the “normal scenes” and the “scrambled objects” sets. Each participant viewed a stimulus two times during each run in a pseudo-random order: First, the “normal scenes” were randomly mixed with either the “cutout objects” (run 1 and 3) or the “scrambled objects” (run 2 and 4) to create a stimulus series. This series was presented twice during a run. All visual stimuli were programmed using the Psychtoolbox (Brainard, 1997), and fed to the projector using an Apple MacBook Pro laptop (2.33 GHz Intel Core 2 Duo processor with 2 GB’s of RAM).

### Eyetracking

Eye movements made during the fMRI experiments were recorded using an MR compatible eyetracker (IviewX MRI) with a temporal resolution of 50 Hz. (SMI, Teltow, Germany). Before commencing the functional runs, calibration of the eyetracking system took place using a nine-point calibration technique. The nine points were placed on a grid covering the central 800 × 600 pixels of the display where the images were displayed. The calibration was validated, and a recalibration was performed when necessary until a good calibration was achieved.

### Functional Imaging

Four runs of 157 BOLD volumes (EPI) each were recorded with a Repetition Time (TR) of 2000 ms, Echo Time (TE) of 28 ms and a flip angle of 70°. Each functional volume contained 39 slices with an in-plane resolution of 64 × 64 pixels. The Field of View was set to 224 × 156 × 224 mm (voxel size: 3.5 × 4 × 3.5 mm). This setting was chosen to allow for recording of the whole brain. Furthermore, an anatomical T1 (Fast Field Echo) scan was recorded (160 slices with a resolution of 256 × 256 pixels). Field of View was 224 × 160 × 224 mm (voxel size: 0.8 × 1 × 0.8 mm).

### Field of view experiment

We tested the influence of the narrow bore of the MR scanner on eye movement parameters in a separate experiment performed outside the scanning environment. For this experiment, a 17″ LCD monitor at a resolution of 1024 × 768 was used. Stimuli were presented a total of four times using two display sizes were used (full screen and half the size of the screen in both dimensions) and two presentation times (10 and 20 s). Stimuli contained all the images from the main experiment and were presented two times in random order within a block of identical presentation size and duration. 15 new participants (10 of whom were female) with healthy vision performed this experiment for which eye tracking was recorded (monocular, right eye) using an Eyelink 1000 (Desktop mount version) at a temporal resolution of 1000 Hz. The order of conditions was balanced across subjects to limit possible effects due to order of presentation. For stability, participants were asked to place their head into a chin rest. We examined the existence of separate viewing modes in eye behavior by plotting fixation duration vs. saccade amplitude as reported in Unema et al. (2005).

## Analysis

Fixations were extracted using IViewX software (SMI, Teltow Germany) with minimum fixation duration set to 80 ms. All subsequent analyses were performed in Matlab 7.4 (Mathworks, Natick MA, USA). Saccadic amplitudes were calculated based on screen positions of subsequent fixations (not separated by blinks), due to the resolution of the eyetracker (50 Hz). Events where blinks occurred in between were filtered out.

Fixations during stimulus presentation were extracted and their durations were plotted against a binned timeline. In total, participants made 11027 fixations during stimulus presentation. For the initial analysis, fixations and subsequent saccades were classified into one out of four categories: Short fixations (< 200 ms) followed by small saccadic amplitudes (< 7.8°, i.e., 250 pixels on screen), short fixations followed by large saccadic amplitudes (>= 7.8°) (“scanning”), long fixation durations (>= 200 ms) followed by small saccadic amplitudes (“inspection”) and long fixation durations followed by small saccadic amplitudes. Cut-off values were data-driven and determined based on the 70th percentile (30% short fixations, 70% long fixations, 30% small saccades, 70% large saccades). The onsets and durations of all fixations in these categories were written to a design file in SPM format. Beforehand, eye movement timings were orthogonalized on the presentation sequence (block design) for each stimulus types (normal scenes and cutout objects, random objects). This orthogonalization was performed to rule out possible effects due to the type of scene (“normal scenes”, “cutout objects” or “scrambled objects”).

### fMRI Analysis

Preprocessing of the functional imaging data was performed in SPM5^1^ in Matlab and consisted of realignment to correct for subject movement, coregistration to align all functional data to the subjects’ T1 image, normalization to convert all images to MNI space. Smoothing was applied using a full width at half maximum (FWHM) of 8 mm. Statistical parametric maps were generated using the design files with the canonical haemodynamic response function.

First, the overall effect of both scanning and inspection types of eye movements were calculated vs. baseline (i.e., the level of brain activity while a white fixation cross was presented on a black screen). A direct comparison of both modes of viewing behavior was constructed using the contrasts “scanning > inspection” and “inspection > scanning”.

### Independent component analysis

We conducted a spatial Group Independent Component Analysis (ICA) of 30 components using the Group ICA of fMRI toolbox version 1.3g (Calhoun et al., 2001) . This number of components was estimated beforehand using the mean value of Minimum Description Length (MDL) across subjects (McKeown et al., 1998; Calhoun et al., 2001). The MDL provides a criterion for the selection of models, regardless of their complexity, without the restrictive assumption that the data form a sample from a “true” distribution. Next, we tested whether any of the components was significantly related to viewing behavior using the following two contrasts: (1) short fixation durations > long fixation durations; (2) small saccades > large saccades and two interaction contrasts; (3) short fixations combined with small saccades > short fixations combined with large saccades; and (4) long fixations combined with small saccades > long fixations combined with large saccadess. Note that for these particular tests, their reverse is equivalent. A component was considered to be significant on the basis of *p* < 0.05, bonferroni corrected.

To further explore the effect of both fixation duration and saccade amplitude on the activity in the significant components, we extracted beta weights (i.e., effect sizes) averaged across each component map. For this, event-related statistical parametric models were built with all fixation events in one regressor and with three parametric modulations; one for fixation duration, one for saccade amplitude and one for the interaction term “fixation duration × saccade amplitude”. This resulted in a total of four beta weights. Finally, a series of *t*-tests were performed to investigate difference in effect size between each pair of significant components.

## Results

### Analysis of viewing behavior

**Figure 2:**
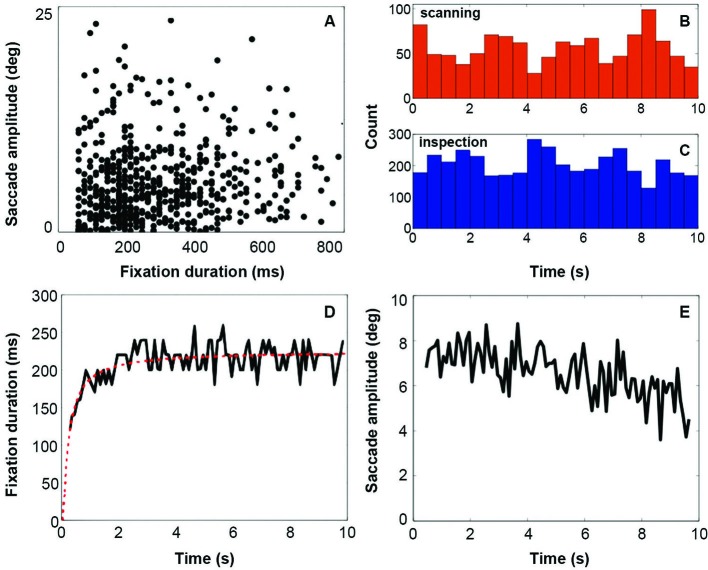
**Eyetracking results**. Panel **A** indicates the distribution of fixations and following saccades plotted in terms of fixation duration against saccade amplitude. Panels **B** and **C** show the distribution of all viewing events (fixation followed by a saccade) of each viewing mode across the display of the stimulus (red = scanning, blue = inspection). Count is the total number of events for all subjects. Panel **D** shows fixation duration over stimulus presentation time with a running average across 50 ms. Panel **E** shows the saccade amplitude over stimulus presentation time with a running average across 50 ms.

Results from the eye tracking recordings (Figure 2) show that fixation duration increases across the 10 s of stimulus presentation (Figure 2, Panel **D**). Initial fixation durations are relatively short. Fixation durations increase rapidly over the first 2 s, and remain relatively constant after that. This behavior is very similar to that reported by Unema et al. (2005). At the same time, saccadic amplitude remains relatively constant over entire duration of the presentation (Figure 2, Panel **E**). This deviates somewhat from that reported by Unema et al. (2005). They described an initially steep decrease in saccade amplitude as a function of the stimulus presentation time. Panels **B** and **C** of Figure 2 show eye movement behavior after categorizing it in terms of the scanning (Panel B) and inspection (Panel **C**) types of behavior. Both types of viewing behavior are encountered approximately equally frequently across the presentation duration of the images. Panel **A** in Figure 2 provides a scatter plot of one examplary individual showing fixation duration vs. saccade amplitude.

### Field-of-view experiment

A possible cause for the difference between our present results and those of Unema et al. (2005) is the relatively small field of view of the display in the MR scanner. To examine the influence of display size, we compared fixation durations and saccade amplitudes for two different field of views. This experiment was conducted outside the MR scanner with different subjects. One display was comparable in size to that used by Unema et al. (2005) (31 × 26°) whereas the second one was comparable to that used in the scanner (25 × 20°).

**Figure 3:**
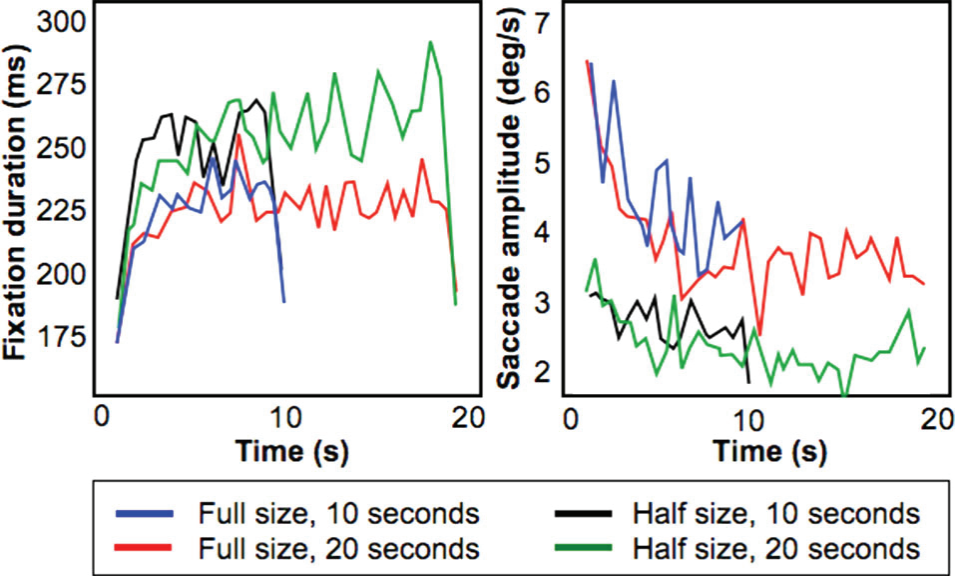
**Results from the Field-of-View Experiment**. Figures show fixation duration duration (left) and saccade amplitude (right) for four conditions (Full/Half size presentation of a stimulus, presentation duration for 10/20 s).

Figure 3 shows fixation duration (left) and saccade amplitude (right) plotted as a function of presentation time using a bin size of 500 ms. These results shows that the increase of fixation duration with presentation time remains present also for relatively smaller stimuli, but that the initial decrease in saccade amplitude is smaller.

To test this, we performed a least squares linear fit within-subject across the 10 s stimulus presentation duration on both fixation duration and saccade amplitude. For saccadic amplitude, we found that for the “Half-size, 10 s” condition the average fitted slope was −0.02° (standard error of 0.0275°), whereas for the “Full-size, 10 s” condition the slope was −0.36° (standard deviation of 0.14°). This difference was significant (*p* < 0.05; paired *t*-test).

For fixation duration, for the condition “Half-size, 10 s” the average fitted slope was 0.25 ms (standard error of 0.225 ms) while for the condition “Full-size 10 s” the average fitted slope was 0.2 ms (standard deviation of 0.275 ms). This difference was not significant. Therefore, this experiment indicates that the smaller decreasing trend in saccadic amplitude inside the MR scanner can be attributed to the limited field of view of the display used.

### fMRI results

**Figure 4:**
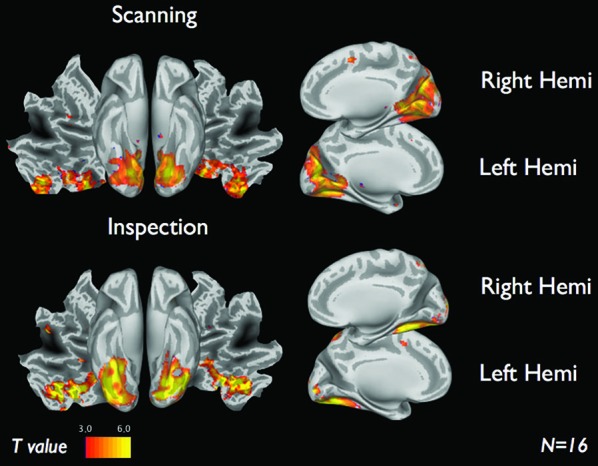
**fMRI results of each viewing mode vs. baseline for 16 subjects**. Scanning (short fixations followed by large saccades) indicate visual regions near the cuneus. Inspection (long fixations followed by small saccades) indicate brain activity along the ventral stream. *Results display T-maps, thresholded with a value of T > 3.*

**Figure 5:**
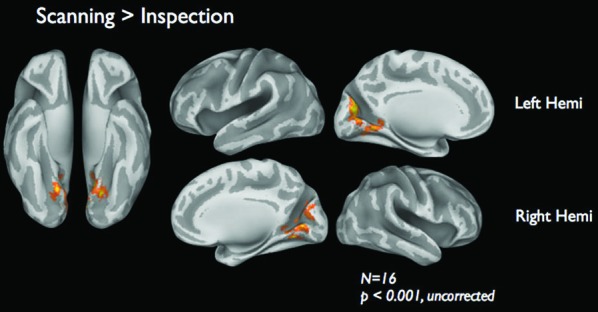
**fMRI results of the direct comparison “scanning” > “inspection”**. Results are based on 16 subjects presented at a lenient threshold of *p* < 0.001, uncorrected.

Figure 4 shows the brain activations for the two categories of viewing behavior when compared against fixation cross (baseline). Scanning behavior, i.e., short fixations followed by large saccades, is correlated with activity that predominates in ventromedial occipital areas. Inspection behavior, i.e., longer fixations followed by small saccades, is correlated with activity in more ventrolateral occipital regions. At first glance, there appears to be little overlap in the regions activated by the two different categories of viewing behavior. Figure 5 shows the statistical parametric map for the direct comparison of the two viewing modes (“scanning > inspection”). This analysis indicates, however, that only at a relaxed threshold, (*p* < 0.001, uncorrected), a statistical differentiation of the two viewing modes in the ventral visual cortex can be demonstrated. The contrast “inspection > scanning” did not reveal significant results.

Standard GLM, as performed above, informs about the activity of certain areas in certain conditions, but not about the degree to which a particular region can be considered to contribute to a network. ICA, on the other hand, will reveal independent and separate networks, that can than be associated with a particular experimental condition or specific behavior. For this reason, we analysed the same dataset again, this time first performing an ICA in order to segregate the brain activity into different components/clusters that can be considered seperate networks. This resulted in 30 components.

**Figure 6:**
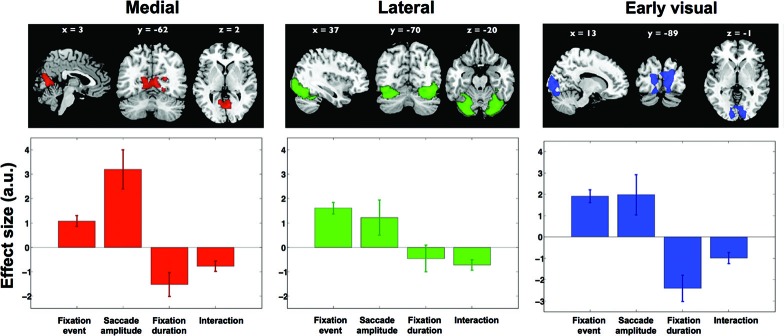
**Results from ICA**. Three independent component maps reflecting visual cortex, and their average beta weights in the fixation duration and saccade amplitude models. Out of 30 components these remained significant with the direct comparison of scanning and inspection. Brain maps were thresholded at *Z* > 2.5. Lower graphs show effect sizes in the parametric GLM for: (1) the main effect of a fixation, (2) saccadic amplitude modulation, (3) fixation duration modulation, and (4) the interaction between fixation duration and saccadic amplitude. Error bars denote standard error of the mean over subjects.

Next, four contrasts were examined to test whether and how activity in each of these components was associated with viewing behavior. Only the interaction term “short fixations and large saccades > long fixations and small saccades” was significant in three of the 30 components. None of the other contrasts reached significance in any of the components. The three components cover distinct regions in visual cortex and are shown in the upper row of Figure 6. The first component (displayed in red, Figure 6) is located in the ventromedial occipital cortex and covers parahippocampal areas. The second component (displayed in green, Figure 6) is located more occipital and ventrolateral and covers the lateral occipital complex. The third component (displayed in blue, Figure 6) covers early visual cortex in particular. The significance of the interaction term “short fixations and large saccades > long fixations and small saccades” indicates that scanning behavior resulted in more activity than inspection behavior throughout early and ventral visual cortex.

To further explore the underlying activity patterns, we extracted average effect sizes for a statistical parametric model with fixation events, two parametric modulations (fixation duration and saccade amplitude) and an interaction term. In Figure 6, the extracted effect sizes are shown below each of the three clusters found in the ICA (upper row). Note that in itself the directions of these findings should not come as a surprise, as this is anticipated based on the significance of the above mentioned interaction term “short fixations and large saccades > long fixations and small saccades”. What is revealed by this analysis though, is the relative magnitude of these effects in the three different clusters.

In the ventromedial cluster (red in Figure 6), the effect size for saccade amplitude is positive and relatively large, confirming that more activity is associated with larger than with shorter saccades. As expected, the effect size for fixation duration is negative, indicating more activity for shorter than for longer fixations.

In the ventrolateral cluster (green in Figure 6), the effect sizes are much smaller than those in the ventromedial cluster. Post-hoc paired *t*-tests between the magnitude of the effect sizes for the three clusters were performed for all conditions and are displayed in Table 1. The effect sizes for fixation event, saccade amplitude and fixation duration differ between the ventromedial and the ventrolateral cluster. For the cluster in early visual cortex (blue in Figure 6), the effect size for fixation event differs from that in the ventromedial cluster, while the modulatory effect of fixation duration differs from that in the ventrolateral cluster. Other effect sizes do not differ from those in the other two clusters. In all three clusters, there is a small negative interaction term, indicating that the modulating influence of saccade amplitude is less for longer fixations than for shorter fixations (Table 2). The magnitude of this interaction effect does not differ between the clusters.

**Table 1:** **Results per condition from all paired *t*-tests performed between the effect sizes of all three components against all other effect sizes**.

**Condition**	**Component 1**	**Component 2**	***P*-value**
**Fixation event**	ventromedial	ventrolateral	0.02*
	ventrolateral	early visual	0.8
	ventromedial	early visual	0.04*
**Saccade amplitude**	ventromedial	ventrolateral	0.0001**
	ventrolateral	early visual	0.3
	ventromedial	early visual	0.15
**Fixation duration**	ventromedial	ventrolateral	0.01*
	ventrolateral	early visual	0.02*
	ventromedial	early visual	0.18
**Interaction**	ventromedial	ventrolateral	0.65
	ventrolateral	early visual	0.67
	ventromedial	early visual	0.74

*p < 0.05, **p < 0.001

**Table 2:** **Results per condition of student’s *t*-tests for each effect size for each cluster significantly different from 0**.

**Condition**	**Component**	***P*-value**
**Fixation event**	ventromedial	0.002
	ventrolateral	0.004
	early visual	0.001**
**Saccade amplitude**	ventromedial	0.001**
	ventrolateral	0.04*
	early visual	0.03*
**Fixation duration**	ventromedial	0.03*
	ventrolateral	0.1
	early visual	0.03*
**Interaction**	ventromedial	0.02*
	ventrolateral	0.03*
	early visual	0.02*

*p < 0.05, **p < 0.001

We also tested whether the effects found in these components could stem from other, picture-related effects. To do so, we analyzed the following contrasts: “normal scenes > cutout objects”, “cutout objects > normal scenes”, “cutout objects > scrambled objects”, “scrambled objects > cutout objects”, “normal scenes > scrambled objects” and “scrambled objects > normal scenes”. None of the tests revealed a significant effect for these components. Therefore, we conclude that the differential activity in the clusters is primarily related to the differences in viewing behavior of the observers.

## Discussion

We report on a functional magnetic resonance study in which we measured the brain activity of 16 observers’ during the free viewing of computer-generated images. Observer’s eye-movements were recorded using a MR-compatible eye tracker. Using a combination of ICA and fixation-based event-related analysis (Marsman et al., 2012), we find that the activity in different regions in the visual cortex is differentially associated with observer’s viewing behavior. Below, we discuss the conclusions we draw from our study, as well as the limitations of our present approach.

### Preattentive and attentive viewing modes do not modulate dorsal visual processing

One of the motivatons for performing this study came from a behavioral eye-movement study by Unema et al. (2005). According to the theory proposed by these authors, dorsal and ventral processing would be associated with distinctive viewing behavior (“pre-attentive” and “attentive” in nature, respectively). Our results do not corroborate this theory. Neither in the GLM-based approach, nor in our ICA-based approach, we found clear evidence of dorsal processing coupled to eye-movements. Our main eye-movement–related activations occured in early visual cortex and the ventral visual cortex.

### Viewing modes in eye tracking data are influenced by display size

Unema et al. (2005) theory about the existence of distinct modes of visual processing was grounded in findings about how eye movement behavior develops as a function of stimulus presentation time. It is therefore important to establish that the viewing behavior we recorded in the scanner environment conformed to this same pattern. Indeed, the pattern of fixation duration in our eye tracking results (Figure 2) was similar to that of Unema et al. (2005), although on average they found shorter initial fixation durations. However, such longer initial fixation durations (approx. 200 ms), as we find now, have also been reported previously (Unema et al., 2005; Hooge et al., 2007). Furthermore, Unema et al. (2005) reported also a decreasing trend for saccadic amplitudes. This initial drop in saccadic amplitude was less clearly visible in our experiment.

To study the origin of this difference, a separate eye tracking experiment using different subjects conducted outside of the scanner indicated that these findings are due to the relatively small size of the stimulus inside the bore of the magnet (see Figure 3). During this experiment, pictures as used in the MRI experiment were shown in two sizes and for two presentation durations. For the smaller images, the decreasing trend for saccadic amplitude was much less distinctive. The initial increase of fixation duration across stimulus presentation was present for both large and small presentations of the images. Unema et al. (2005) used a smaller cut-off value to determine ambient and focal viewing modes for saccadic amplitude. We used a data-driven approach in which the 70th-percentile of the saccades was defined as the cut-off value 30% small saccades, 70% large saccades). This is the reason that we employed a different cut-off value for our saccadic amplitude in the MR experiment in comparison to Unema et al. (2005).

Based on the significant difference between fitted slopes of the saccadic amplitude curves for the “Half-size” and the “Full-size” conditions, we conclude that despite the smaller display size facilitated by the scanner environment and a smaller number of different stimuli used, our observers’ viewing behavior conformed to the patterns described by Unema et al. (2005).

### Three independent components in visual cortex are associated with viewing behavior

We chose to explore the use of blind source separation (ICA), as it has been proven to be very suitable for studying natural viewing in fMRI (Bartels and Zeki, 2004; Malinen et al., 2007). Using such blind source separation methods we find evidence for three separate components that are related to our measures of viewing behavior, of which one component is situated in primary visual cortex and two in the ventral cortex (Figure 6).

### Pre-attentive viewing modulates activity in early visual and ventromedial cortices

The GLM-based analysis indicates that the main difference between activity associated with different viewing modes could be found in ventromedial cortex. However, the effect was not very strong and could only be retrieved when applying a relatively lenient statistical threshold (Figure 5). Nevertheless, the ICA approach corroborated that the ventromedial cluster in particular is modulated by eye movement characteristics. Activity in this cluster (red in Figure 6) was significant and positively modulated by saccade amplitude and negatively by fixation duration. The processing in this region therefore appears to be most clearly associated with the “preattentive”, “ambient”, or “scanning mode” viewing behavior as defined by Unema et al. (2005) (short fixations in combination with large saccades). Activity in the cluster in the ventrolateral visual cortex (green in Figure 6) was much less distinctively modulated by any of the eye-movement characteristics considered. In both other clusters, the modulatory influence of fixation duration was larger. The modulatory influence of saccade amplitude was similar to that in the visual cortex cluster, but much smaller than that in the ventromedial cluster.

Previous studies on scene perception suggest that during the early stages of perception, a schematic representation of the scene is captured, which subsequently guides eye movements (Rensink, 2004). This initial representation is commonly referred to as the “gist” of a scene (Torralba et al., 2006). Presently, regions in the ventromedial cortex are assumed to be involved in generating the gist of a scene (Fize et al., 2000). Furthermore, several studies have investigated the nature of this mechanism and propose that it is based on extracting global statistical features (Cant et al., 2009; Cornelissen et al., 2009). In parallel, behavioral studies have shown that the average fixation duration of viewing behavior increases as a function of stimulus presentation time (Antes, 1974; Friedman and Liebelt, 1981; Unema et al., 2005; Hooge et al., 2007). This indicates that early stages of perception involve brief fixations coupled with large saccadic eye movements. Unema et al. (2005) proposed that this early viewing behavior represents a pre-attentive or “ambient” mode of perception. During this ambient mode, the dorsal pathway was hypothesized to be mostly active, when it deals with layout of objects in the scene. However, in contrast with this hypothesis that predicts more parietal activity, we find predominantly ventromedial activity for this type of viewing. This could imply that during such scanning behavior, information is processed at a statistical level, where—in line with findings in the scene perception literature—global features are extracted. In turn, this suggests that the visual system may comprise two types of processing, the activity of which is associated to the eye movements we make.

### Does eye-movement related cortical activity reflect top-down or bottom-up processing?

Eye-movements not only depend on bottom-up components of processing, but will also be associated with top-down processing related to saccade-planning and determining currently required task-relevant information (Ballard and Hayhoe, 2009). As such, we believe that it is most likely that our current activity patterns integrate activity of both top-down and bottom-up processing components. For this reason, it is also unlikey that each fixation and saccade would initially have the same neural activity map that only starts to deviate after a particular time. The use of imaging modalities with higher temporal resolutions could perhaps give a more detailed insight in the spreading of activation throughout the visual system following a fixation. In our experiments, participants were performing natural viewing behavior. However, when specific task instructions would be given, we would expect to find different patterns of viewing behavior (conform the earlier results of Yarbus (1967)).

### Limitations of the present study

In both the “Field-of-View” and the fMRI experiments, we presented each stimulus more than once. This could have influenced both the eye movement patterns as well as perception over time, and, consequently, may have affected the fMRI signal as well. Another limitation in the current paradigm is that participants viewed static computer-generated stimuli for 10 s. Future experiments could therefore improve on the present paradigm by examining viewing behavior in dynamic, natural stimuli.

## Conclusion

We started the present experiment, expecting that activity patterns associated with different types of viewing behavior would reveal dorsal and ventral visual regions in the human brain. We do not find this. Further exploratory analyses revealed that eye movement behavior consisting of short fixations and large saccades (“scanning behavior”) in particular is associated with activity in a ventromedial occipital region. This corroborates with the current understanding of the involvement of this region in fast “gist-based” scene perception. Ventrolateral parts in visual cortex, currently understood to be involved in (detailed) shape and object recognition, was much less affected by the specific eye-movement parameters. Eye-movement characteristics thus differentially influence neural processing in different regions in visual cortex. In summary, we conclude that evaluating the modulatory influence of viewing behavior is crucial for unraveling natural cortical visual processing.

## Conflict of interest statement

The authors declare that the research was conducted in the absence of any commercial or financial relationships that could be construed as a potential conflict of interest.
